# Glycosphingolipid Levels in Urine Extracellular Vesicles Enhance Prediction of Therapeutic Response in Lupus Nephritis

**DOI:** 10.3390/metabo12020134

**Published:** 2022-02-01

**Authors:** Brian Troyer, Jessalyn Rodgers, Bethany J. Wolf, James C. Oates, Richard R. Drake, Tamara K. Nowling

**Affiliations:** 1 Department of Medicine, Rheumatology Division, Medical University of South Carolina, Charleston, SC 29425, USA; troyerb@musc.edu (B.T.); ierardi@musc.edu (J.R.); oatesjc@musc.edu (J.C.O.); 2 Department of Public Health Sciences, Medical University of South Carolina, Charleston, SC 29425, USA; wolfb@musc.edu; 3 Rheumatology Section, Ralph H. Johnson VA Medical Center, Charleston, SC 29403, USA; 4 Department of Cell and Molecular Pharmacology and Experimental Therapeutics, Medical University of South Carolina, Charleston, SC 29425, USA; draker@musc.edu

**Keywords:** lupus nephritis, biomarker, glycosphingolipid, extracellular vesicle

## Abstract

The development of nephritis increases the risk of morbidity and mortality in systemic lupus erythematosus (SLE) patients. While standard induction therapies, such as mycophenolate mofetil (MMF) induce clinical remission (i.e., complete response) in approximately 50% of SLE patients with nephritis, many patients fail to respond. Therapeutic response is often not assessed until 6–12 months after beginning treatment. Those patients that fail to respond to treatment continue to accumulate organ damage, thus, there is a critical need to predict which patients will fail therapy before beginning treatment, allowing physicians to optimize therapy. Our previous studies demonstrated elevated urine, but not serum, glycosphingolipids (GSLs) in SLE patients with nephritis compared to SLE patients without nephritis, suggesting the urine GSLs were derived from the kidney. In this study, we measured the GSLs hexosylceramide and lactosylceramide in extracellular vesicles isolated from longitudinal urine samples of LN patients that were treated with MMF for 12 months. GSL levels were significantly elevated in the baseline samples (prior to treatment) of non-responders compared to complete responders. While a few other proteins measured in the whole urine were higher in non-responders at baseline, only GSLs demonstrated a significant ability to discriminate treatment response in lupus nephritis patients.

## 1. Introduction

Systemic lupus erythematosus (SLE) is a type III hypersensitivity autoimmune disease that can affect many different tissues and organs. Lupus nephritis (LN), an immune complex-mediated glomerulonephritis, affects up to two-thirds of SLE patients [1]. Although there are newly approved treatments for LN that seem promising [2,3], treatment of LN is often based on immunosuppression and less on targeted immunomodulation. Despite standard treatment protocols, progression of the most aggressive form of LN (class III and IV) to end-stage renal failure remains high [4]. Thus, there is a need for biomarkers of therapeutic response that would allow physicians to make better-informed treatment decisions for LN patients. Parameters determining response are often conventional markers, such as serum complement C3 (C3), albumin, serum creatinine, and urine creatinine, eGFR, urine protein, and urine protein to creatinine ratio (UPr:UCr). Although these parameters are useful, they often require at least six months for evaluation of treatment response, and long-term survival is heavily predicated on early detection of treatment response [5].

These conventional markers have limited utility for predicted relapse and overall disease activity. Due to these shortcomings, renal biopsy remains the gold standard for detection, the extent of disease, and therapeutic response [6] at the expense of cost and invasiveness. Abundant research into novel urine biomarkers of disease or therapeutic response was published over the past decade [7,8,9,10]. Urine, as a direct product of the kidneys, likely reflects disease activity of LN. Within urine, extracellular vesicles (EV) such as exosomes, are also being investigated as sources of potential novel biomarkers. Since EVs contain molecules of the cells from which they were derived, urine EVs likely contain molecules, including glycosphingolipids (GSLs), derived from renal cells and may contain markers of disease activity or damage [11]. We previously demonstrated that GSLs are elevated in the urine or kidney of lupus patients and lupus mice with nephritis [12,13]. However, to our knowledge, no studies have investigated urine GSLs as potential biomarkers of therapeutic response.

Many cellular processes, such as cell signaling, proliferation, and inflammation, are modulated by GSLs, often by serving as receptors for pathogens, and are abundant in the kidney [14]. GSL metabolism was shown to be altered in several kidney diseases [15,16], including LN [12]. GSLs consist of a ceramide backbone and the type and number of sugars added to ceramide generate a broad variety of GSLs. The GSL glucosylceramide (GlcCer) is generated by the addition of glucose to ceramide, which can then be converted to lactosylceramides (LacCers) by the addition of a galactose residue. Hexosylceramides (HexCers) are comprised of GlcCers and galactosylceramide (GalCers), which are difficult to separate. We previously showed that the elevated HexCers in urine and kidney of lupus mice with nephritis was due to increases in GlcCers and not GalCers as measured by SFC-MS/MS [12]. GSLs are further distinguished by the fatty acid chain length.

Based on our previous observations, we tested if GSLs in urine EVs are potential biomarkers of therapeutic response. We show that GSLs are significantly elevated in EVs isolated from baseline (pre-treatment) urine samples of LN patients that failed to respond to treatment compared to LN patients that completely responded to treatment with mycophenolate mofetil (MMF). Moreover, these GSLs demonstrated good predictive ability to discriminate between non and complete responders with areas under the receiver operating characteristic curve of >0.8. While several additional proteins in baseline whole urine in this same population of patients were also significantly elevated in non-responders, none could discriminate between non and complete responders. Thus, GSLs may serve as novel predictive biomarkers of therapeutic response prior to beginning treatment in LN.

## 2. Results

### 2.1. Glycosphingolipids in Urine Extracellular Vesicles May Serve as Biomarkers to Predict Therapeutic Response

Longitudinal urine samples were obtained from 58 LN patients from the three cohorts (LUNAR, Abatacept, MUSC) that had urine available at 0 (baseline), 3, and/or 12 months. Baseline and one-year clinical/response data were also collected. Baseline samples from 4 of the 58 patients were exhausted in pilot studies to test workflow and to perform a “discovery” proteomics screen (see Figure S1 for sample workflow). Demographic and baseline clinical characteristics of the remaining 54 patients, whose samples were included in the full study, are presented in Table 1. Baseline UPr:UCr, eGFR, and serum creatinine differed between complete responders (CR) and non-responders (NR). Specifically, responders had significantly higher baseline eGFR and lower UPr:UCr and serum creatinine (*p* < 0.01 for all comparisons). The racial distribution differed between CR and NR such that a significantly greater proportion of treatment CR was black compared to NR, while a significantly greater proportion of NR was Hispanic/other compared to CR. No differences were noted in age, C3 complement, C4 complement, or dsDNA levels. A majority of the patients had pure proliferative (class III or IV) nephritis. Since most of the samples were obtained from two independent cohorts (LUNAR and abatacept clinical trials), we also analyzed potential clinical and demographic differences between these cohorts. The only difference was in racial distribution.

**Figure 1:**
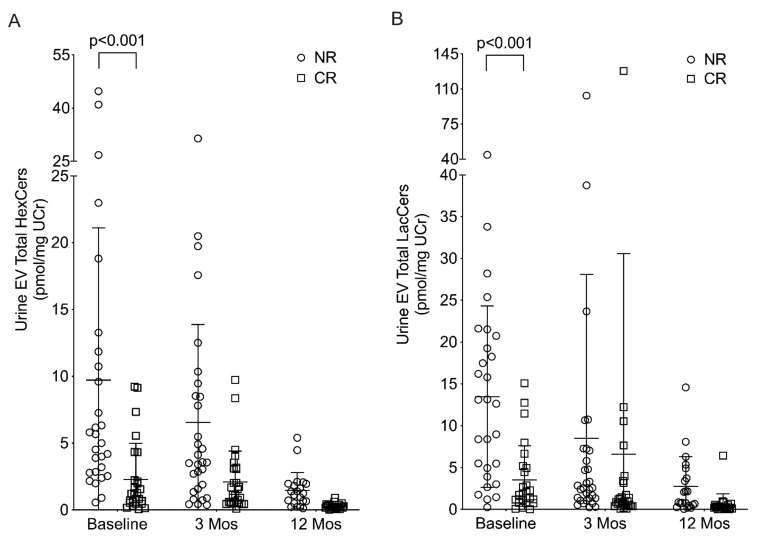
Total HexCers and LacCers are significantly higher in baseline urine EVs of patients that did not respond to therapy. EVs were isolated from urine samples of LN patients that met the clinical criteria of a non-responder (NR) or a complete responder (CR) after 12 months of treatment with MMF. Total of all chain lengths of HexCers (**A**) and LacCers (**B**) were quantified in EVs from urine collected prior to treatment (baseline) and at 3 months (3 Mos) and 12 months (12 Mos) post-treatment. HexCers and LacCers were normalized to urine creatinine (UCr) levels measured in the urine samples from which the EVs were isolated. *p*-values were calculated as described in the Methods and adjusted for multiple comparisons using Bonferonni correction. Adjusted *p*-values are provided on the graph.

All chain lengths (C14 to C24) of HexCers and LacCers were quantified in the EVs isolated from baseline (prior to treatment) and at 3 and 12 months post-MMF treatment urine samples. Total HexCers (Figure 1A) and total LacCers (Figure 1B) normalized to urine creatinine (UCr) were significantly higher in the NR group compared to the CR group prior to beginning treatment (baseline). The levels in both groups decreased from baseline to 12 months. Differences between CR and NR in GSLs at baseline, 3 months, and a change from baseline to 3 months were considered but the only significant differences were observed at baseline. While these GSLs may not be useful to determine response at 3 and 12 months, their changes may be useful for ensuring a continued response to treatment. Similar results were observed between NR and CR at baseline when the GSL levels were normalized to inorganic phosphate (Supplementary Figure S2) or total protein content measured by pyrogallol (data not shown).

The significant differences between NR and CR remained when examining the individual chain lengths of HexCer and LacCer normalized to UCr (Supplementary Figure S3A,B). Univariate tests demonstrate that NR had significantly higher levels of all HexCer and LacCer chain lengths at baseline after adjusting for multiple comparisons (Table 2). Normalizing levels to EV inorganic phosphate levels resulted in similar differences between NR and CR (Supplementary Figure S3C,D) although the significant differences were lost after adjusting for multiple comparisons. To ensure that the LN class did not account for the significant differences observed, the UCr normalized data were analyzed after removing patients with LN classes I and V, or no biopsy (Table S1) or after removing patients without pure LN class III or IV (Table S2). These analyses demonstrated that the differences between NR and CR in total HexCers and total LacCers, as well as most of the individual chain lengths, remained statistically significant.

Using data from all samples in Table 1, individual chain lengths and total HexCers or LacCers levels demonstrated good predictive ability to discriminate between non and complete responders with areas under the receiver operating characteristic curves (AUCs) >0.84 in multivariate logistic regression models adjusted for baseline UPr:UCr and eGFR (Table 3). The associations remained significant after adjusting for multiple comparisons for most individual and total HexCers, and for most individual and total LacCers. Models including only baseline eGFR, UPr:UCr, or both eGFR and UPr:UCr had AUCs of 0.73, 0.74, and 0.77, respectively. This suggests that the inclusion of GSLs improved the ability of the models to discriminate between NR and CR showing an increase in area under the receiver operating characteristics curve (AUC) between 0.07 and 0.12 relative to the model excluding GSLs (Table 3).

### 2.2. Urine Proteins Elevated in Non-Responders, but May Not Predict Therapeutic Response

**Figure 2:**
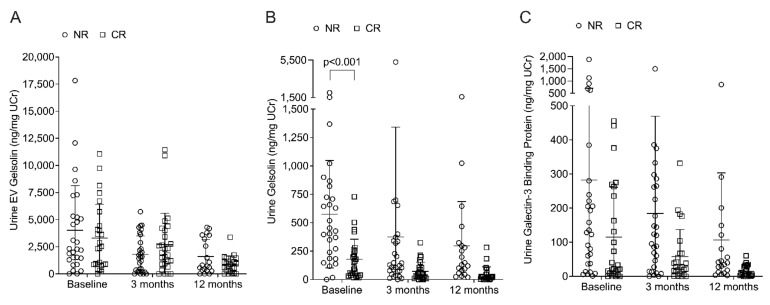
Gelsolin is significantly higher in baseline urine of patients that did not respond to therapy. Gelsolin was quantified in urine EVs (**A**) and whole urine (**B**) for all available LN patients (see Figure S1). (**C**) Galectin-3 binding protein was measured in whole urine of the same patients. All measures were normalized to UCr. *p*-values were calculated as described in the Methods and Bonferonni adjusted *p*-values are provided.

In addition to GSLs, a proteomics analysis was performed on a subset of urine EV samples (5 CR and 5 NR, see Figure S1) to identify potential novel proteins of therapeutic response. For the proteomics, these 10 EV samples were further processed to remove albumin to enhance detection of less abundant proteins. Although albumin remained the most highly abundant protein in the EVs, additional proteins of interest were detected (Figure S4). Among the proteins that were readily detected, we chose to further examine proteins previously identified as potential biomarkers of LN (prostaglandin-H2 D-isomerase and galectin-3 binding protein) and proteins not previously identified as potential LN biomarkers but shown to function in the kidney (Cornulin and Gelsolin). Prostaglandin-H2 D-isomerase (PTGDS) was difficult to detect in the EVs with most samples below the standard curve on the individual ELISA. Galectin-3 binding-protein, also known as lectin galactoside-binding soluble 3-binding protein (LGALS3BP), and a known biomarker of LN [7,17], was detected in both EVs and whole urine. Cornulin (CNN) was undetectable in both EVs and whole urine, while gelsolin (GSN) was detectable in both EVs and whole urine in individual ELISAs. Due to limited EV samples, we chose to measure gelsolin in both EVs and whole urine and LGALS3BP in whole urine only. Gelsolin levels in the EVs did not differ by response status (Figure 2A) at any time point. In whole urine, gelsolin (Figure 2B) and LGALS3BP (Figure 2C) were higher in NR compared to CR at baseline; however, the difference was only significant for gelsolin (*p* < 0.001). As with the EV GSL levels, EV, and whole urine gelsolin levels and whole urine LGALS3BP levels decreased from baseline to 3 months, but the differences were not significant. As with the GSL data, the gelsolin, and LGALS3BP data were also analyzed after removing patients with class I or V LN, or with no biopsy (Table S1), or after removing patients without pure class III or IV (Table S2). Although there was an increase in the median EV gelsolin levels in CR compared to NR after removing the indicated patients, the differences were not significant. The significant difference in whole urine gelsolin between NR and CR remained, regardless of LN class inclusion.

Since chemotaxis pathways are strongly active especially in proliferative LN, we then screened whole urine baseline and 3-month samples to determine if there were urine chemokines that were either differentially present at baseline or were specifically decreased at 3 months post-treatment in the CR group compared to the NR group. For this analysis, a subset of the LUNAR and abatacept patients that had pure class III or IV LN were included (13 NR, 12 CR). In a panel of 23 chemokines, 15 had detectable levels in a majority of the samples and were evaluated for associations with a response status. In univariate analyses, IL-16, LIF, IL-33, IL-23, CTACK, and TSLP were associated with response in patients with LN, but none of the chemokines retained statistical significance after adjusting for multiple comparisons (Table 4 and Supplementary Figure S5). In all cases, higher median levels of the chemokines were observed in NR compared to CR. In multivariable logistic regression models adjusting for baseline eGFR and UPr:UCr, none of the chemokines demonstrated significant ability to discriminate between CR and NR. In addition, none of the chemokines were significantly different between CR and NR at 3 months (Figure S5) or significantly decreased from baseline to 3 months in either group.

## 3. Discussion

The rates of complete remission in LN remain low regardless of therapy. Future treatments for LN (especially classes III and IV) will likely require more targeted approaches and identifying markers that predict response will better inform treatment decisions and improve response rates. We previously showed glycosphingolipid (GSL) metabolism is altered in lupus patients and several lupus mouse strains with nephritis. Specifically, elevated urine lactosylceramide (LacCer) levels were observed prior to proteinuria in lupus mice [12,13,18]. These results suggest urine GSL may serve as potential biomarkers of nephritis and treatment response, and that GSL metabolism plays a role in the pathogenesis of disease. In LN patients, LacCer levels were elevated in urine and not in the serum, suggesting GSL urine levels are largely reflective of renal levels. In this study, we demonstrated that total GSLs hexosylceramides (HexCers) and LacCers in EVs isolated from urine samples collected prior to treatment were significantly higher in LN patients who failed to meet complete response criteria compared to those who had a complete response. We observed in this study that all chain lengths from C16 to C24 of HexCers and LacCers were significantly higher in NR compared to CR.

Our previous studies of urine GSLs were performed using whole urine [12] and here we isolated extracellular vesicles (EVs) from whole urine. EVs are secreted from cells, range in size from 30–1000 nm, and contain receptors, proteins, genetic material, and lipids specific to the cells from which the EVs were derived. Since urine EVs are likely derived largely from the kidney, they may more accurately reflect renal disease activity and response to therapy than levels in whole urine. While nucleic acids, such as microRNA and a few proteins in urine EVs were identified as attractive markers of renal disease in SLE patients [19], GSLs have not been examined. Future studies are aimed at comparing GSL levels in urine EVs to whole urine and to determine which best reflects levels in the kidney.

Importantly, the GSL levels in the urine EVs remained significantly associated with treatment response status in multivariate logistic regression models after adjusting for baseline urine protein to urine creatinine ratio (UPr:UCr) and estimated glomerular filtration rate (eGFR), which were also significantly different between the NR and CR groups. Specifically, association of C20, C24:1, and total HexCers remained significant after adjusting for multiple comparisons. The multivariable models adjusting for UPr:UCr and eGFR suggest that the inclusion of GSLs improved the ability of the models to discriminate between NR and CR showing an increase in the area under the receiver operating characteristics curve (AUC) between 0.07 and 0.12 relative to the model excluding GSLs. Proteinuria (UPr:UCr) was shown in a recent study using multivariate models to be an independent predictor of complete response [20]. Inclusion of UPr:UCr with six other clinical/histological measures resulted in an AUC of >0.75 in five different models [21]. Together, these analyses suggest that urine EV HexCer levels may improve the ability of current clinical/histological measures to predict response to MMF prior to beginning treatment in LN patients.

Since urine EVs also may include cytokines, chemokines, enzymes, vascular molecules, and other proteins that reflect renal pathology, we performed a pilot proteomics screen using a subset of EV samples (5 from NR and 5 from CR). Among the proteins identified in the screen, we examined gelsolin and galectin-3 binding protein (LGALS3BP). Gelsolin was present at higher levels in the CR compared to the NR samples in the proteomics screen. Gelsolin is an actin-binding protein and extracellular gelsolin is thought to be recruited to sites of inflammation to clear actin from damaged tissues. In SLE patients, plasma gelsolin levels were reported to be decreased compared to controls, negatively associated with disease activity, and to be further decreased during a flare [22,23]. Glomerular and tubular gelsolin was detected in lupus patients with nephritis and showed some association with LN classification [24]. Localization of gelsolin to the kidneys was also demonstrated in IgA nephropathy patients and these levels correlated with renal fibrosis while plasma gelsolin in these same patients was decreased [25]. Although our pilot study suggested that urine EV gelsolin levels may be higher in LN patients that were CR compared to NR, the results from the full study showed similar levels in both groups. Gelsolin levels in whole urine were significantly lower in CR compared to NR baseline samples. The whole urine levels of gelsolin may reflect the extracellular levels of renal gelsolin, while the urine EV levels may reflect cellular levels of gelsolin. However, this hypothesis remains untested. Although the whole urine levels of gelsolin were significantly lower in the CR group at baseline, the gelsolin levels did not significantly improve the ability to discriminate between CR and NR.

LGALS3BP protein was not differentially expressed in the urine EV pilot screen. Since the EV elution buffer interfered with the LGALS3BP ELISA, we measured LGALS3BP in whole urine. A recent systematic review of 25 different studies using mass-spectrometry-based proteomics identified approximately 241 candidate biomarkers, including LGALS3BP [7]. LGALS3BP promotes cell-to-cell adhesion and initiates pro-inflammatory signaling and may be a potential biomarker due to its proposed role in LN pathophysiology. Nielsen et al., [17] demonstrated LGALS3BP co-localized with immune complexes deposited in renal glomeruli, was increased in patients with LN compared to healthy controls and had higher levels in patients with active LN. Based on previous studies and our pilot proteomics screen, we anticipated that urine LGALS3BP levels would be similar in both groups and serve as a type of positive control in our study. Indeed, although LGALS3BP urine levels tended to be lower in the CR group compared to the NR group at all time points, the differences were not statistically significant.

Prior research showed that plasma chemokines were significantly elevated in lupus patients with active disease [26] and that chemotaxis pathways were enriched in the urine proteome of LN patients [27]. Our screen of 23 chemokines in a subset of patients showed that IL-16, IL-33, IL-23, CTACK, and TSLP were significantly higher in NR patients at baseline. However, this significance was lost after adjusting for multiple comparisons. These chemokines also did not significantly discriminate between CR and NR and were no different in the extent of their reduction between baseline and 3 months. Overall, these results reflect the broad range of changes in protein levels among patients, with most patients experiencing a significant reduction in levels in response to treatment regardless of response as assessed at 12 months post-treatment.

The strengths of this study included the use of GSL measures and urine EVs, a novel approach for identifying potential therapeutic response biomarkers, and the use of longitudinal samples; however, there were a few limitations. The presence of albumin was a confounder in the identification of proteins in urine EVs and whole urine, as it can mask less abundant proteins in multiple analyses. Large amounts of albumin also resulted in clumping of the EVs, making accurate measures of EV numbers difficult. While removing most albumin allowed for more accurate estimation of EV numbers and identification of less abundant proteins by proteomics, removing albumin is technically time-consuming and may result in degradation/loss of proteins or lipids of interest. However, significant differences observed in the EV data were conserved across several normalization approaches, adding to the confidence in the UCr normalized results. Another limitation of this study was the small samples size of ~30 CR and ~30 NR LN patients, and the evaluation of chemokines included only a subset of those 60 patients. Despite the small sample size, we demonstrated that HexCers enhanced the ability to discriminate between CR and NR prior to beginning treatment and strongly suggest this GSL could be used to predict who will fail treatment. Screening of a larger population of patients is needed to confirm our results.

## 4. Materials and Methods

### 4.1. Ethics Statement and Human Samples

This study analyzed stored longitudinal urine samples collected from SLE patients with nephritis who were treated with mycophenolate mofetil (MMF) for 12 months in two unrelated clinical trials, rituximab in lupus nephritis (LUNAR) [28] and abatacept [29] in lupus nephritis, or SLE patients from the MUSC lupus clinic treated with MMF. All volunteers provided informed consent for their samples to be used for research purposes and the study was approved by the MUSC Institutional Review Board and by the Department of Defense Human Research Protection Office. All patients met the American College of Rheumatology classification for SLE with biopsy-confirmed nephritis of which 93% were classified as class III or IV. Patients were evaluated by treating physicians and identified as complete responders (CR) or non-responders (NR). Criteria for CR included UPr:UCr < 0.5, normal sCr, UPr:UCr reduced > 75%, and eGFR increased > 25%. NR criteria included persistent UPr:UCr > 3, decreased UPr:UCr < 25%, eGFR decreased > 25%, and failure to taper glucocorticoid therapy to <10 mg/day. Samples included: 1) baseline (prior to treatment) and 3 months post-treatment urine from 15 patients (6 CR and 9 NR) in the LUNAR clinical trial [28] who were in the placebo group (MMF + placebo); 2) baseline, 3 months post-treatment, and 12 months post-treatment urine from 37 patients (16 CR and 21 NR) in the abatacept clinical trial [29] who were in the placebo group (MMF + placebo); and 3) baseline, 3 months post-treatment, and 12 months post-treatment urine from 7 patients (all CR) from the MUSC lupus clinic. All patients enrolled in the LUNAR and abatacept trials had class III or IV LN, and 3 of the 7 MUSC clinic patients had class III or IV LN. MMF was given at 2–3 g/day in the abatacept trial by day 15 [29] or 3 g/day in the LUNAR trial by day 16 [28]. More specifics with respect to treatment regimens and dosages for the two trials are provided in the references indicated. Analyses were performed without or with the non-class III or IV LN patients from MUSC, as described in the Section 2. Patient demographics and clinical measures are provided in Table 1.

### 4.2. Urine Extracellular Vesicle Isolation

Urine extracellular vesicles (EVs) were isolated from human urine using Ymir Genomics’ EV Isolation Kit according to manufacturers’ protocols (Ymir Genomics, Cambridge, MA, USA). Aliquots of human urine were centrifuged 2000× *g* for 5 min at 4 °C to remove cell/debris for analyses. Aliquots of whole, debris-free urine were removed and stored at −80 °C until use in ELISAs and a cytokine screen (see below), and 10 mL of debris-free urine was used to isolate EVs, which were aliquoted and stored at −80 °C until use. The recovery and size of EVs were assessed by multiple methods and a proteomics screen was performed in a subset of 10 samples; 5 CR and 5 NR who were all white females with class IV LN only (see Figure S1 for workflow). The methods to assess recovery or size of EVs included the Zetaviewer NTA Exosome Analyzer S/N 17-313 and Zetaview Software 8.04.02 with a 0.743 µm/px camera, a CD63 ExoELISA-Ultra CD63 assay (System Biosciences, Palo Alto, CA), and the Bradford or QuanTest Red Pyrogallol Red Reagent (Quantimetrix, Redondo Beach, CA) protein quantification assays. Due to the high levels of albumin, the Evs tended to clump and interfered with accurate estimation of EV numbers by the Zetaviewer and CD63 ELISA. Several approaches were used to remove albumin. No approach successfully removed all albumin; however, removing most of the albumin using DTT, heat, and ultracentrifugation resulted in significantly less EV clumping and a more accurate assessment of EV numbers by the Zetaviewer that corresponded with CD63 measures. Removing a majority of the albumin required extensive manipulation of the samples, resulting in low yields of EVs. We determined that the pyrogallol protein estimations in the unmanipulated EV samples more accurately reflected the EV estimations in the DTT-treated samples measured by CD63 and Zetaviewer compared to the Bradford protein estimations. Thus, to avoid over-manipulating the samples, the pyrogallol assay was used to assess EV content in unmanipulated samples that were used for measuring EV protein content by ELISA and EV lipid content. Lipid and gelsolin measures in the EVs are presented in the manuscript normalized to UCr. Lipid levels were also examined after normalizing to inorganic phosphate, and gelsolin levels were also normalized to total protein (by pyrogallol) for gelsolin to confirm the results of UCr normalized results.

### 4.3. Lipidomic Analyses

Levels of individual chain lengths C16, C18, C18:1, C20, C20:1, C22, C22:1, C24, C24:1, C26, and C26:1 for GSLs hexosylceramides (HexCers) and lactosylceramides (LacCers), and levels of inorganic phosphate (Pi) were quantified in isolated EVs by the Medical University of South Carolina Lipidomics Core Facility, as previously described [12]. Levels of the major chain lengths expressed and the total of all chain lengths for each GSL are presented normalized to UCr. Levels were also normalized to EV Pi levels and are presented as Supplementary Materials. A subset of 10 baseline samples was randomly selected (5 CR and 5 NR) for a proteomics screen for which some of the samples were exhausted, leaving 28 NR and 26 CR baseline samples, 30 NR and 28 CR 3-month samples, and 21 NR and 23 CR 12-month samples for the EV analyses.

### 4.4. Protein Analyses

Individual ELISAs were used to measure gelsolin (DevKit Duo, Lifespan Biosciences, Seattle, WA) or galectin-3 binding protein (G3BP/MAC-2BP, R&D systems, Minneapolis, MN) in debris-free whole urine according to the manufacturers’ instructions. A modified protocol of the gelsolin ELISA to include EV elution buffer from the Ymir EV Isolation Kit was used to quantify gelsolin levels in the EV samples. A panel of 23 cytokines was measured in debris-free whole urine in a subset of LUNAR and abatacept patients with pure class III or IV LN (13 NR, 12 CR) by Eve Technologies (Human Cytokine/Chemokine 23-Plex Discovery Assay HD23, Calgary, AB, Canada). Levels of all proteins were normalized to urine creatinine (UCr), which was measured using the Jaffé picric acid method.

### 4.5. Statistical Analyses

Descriptive statistics were determined for patient demographics and baseline clinical measures across all patients and by treatment response status across all cohorts and within the cohort. Univariate associations of clinical and demographic characteristics with treatment response status across all cohorts and within the cohort (LUNAR versus abatacept) were evaluated using a series of t-tests or Wilcoxon rank-sum tests for continuous variables and chi-square or Fisher’s exact tests for categorical variables.

The primary goal was to examine associations between lipid and protein levels at baseline with treatment response at one year. Univariate associations between glycosphingolipid (GSL) levels, chemokine levels, and protein levels with treatment response were evaluated using a series of Wilcoxon rank-sum tests. Multivariable logistic regression models adjusting baseline urine protein:creatinine ratio (UPr:UCr) and eGFR evaluating the association between individual GSLs and chemokines were also considered. Likelihood ratio tests (LRT) were conducted, comparing a model with only UPr:UCr and eGFR to a model including UPr:UCr, eGFR, and a specific GSL, chemokine/protein to evaluate the associations after controlling for UPr:UCr and eGFR. For the multivariable logistic regression models, GSL, and chemokine levels were log2 transformed to meet model assumptions. The area under the receiver operating characteristics curves (AUCs) was estimated from the multivariable logistic regression model to describe the ability to discriminate treatment response. Due to the small sample size, the logistic regression model was limited to no more than three predictors [30]. UPr:UCr and eGFR were selected since they are typically used to monitor responses and were also different between NR and CR at baseline. The AUCs were estimated using 5-fold cross-validation for better generalizability [31]. For both univariate and multivariable results, *p*-values were Bonferroni adjusted to account for multiple comparisons and provided as indicated in all tables and figures. All analyses were run in SAS v. 9.4.

## 5. Conclusions

GSLs in urine EVs and all proteins in whole urine were decreased from baseline to 12 months post-treatment in LN patients that both completely responded or failed to respond to mycophenolate mofetil based on the response criteria indicated. The GSLs HexCers and LacCers in urine EVs, and gelsolin, IL-23, IL-16, IL-33, CTACK, and TSLP were significantly higher at baseline in the non-responding patients. Importantly, HexCers enhanced the prediction of therapeutic response, with HexCer C20, HexCer 24:1, and total HexCers having an AUC of 0.89–0.90 after adjusting for eGFR and UPr:UCr. Urine HexCers may serve as potential biomarkers to predict response in LN patients. Furthermore, the elevated levels of HexCers and LacCers suggest GSL metabolism plays a role in pathogenic responses in the kidney and may serve as therapeutic targets. Alternatively, increased renal GSL metabolism may be a response to injury and merely reflect the extent of systemic or renal inflammation or injury. Future studies to include measures of inflammatory burden with respect to the association of GSL levels with therapeutic response are of interest and remain to be determined using a larger sample size. Regardless, measures of GSL levels prior to beginning treatment could enhance the identification of LN patients that require more aggressive/alternative therapy.

## Figures and Tables

**Table 1:** Patient demographics and baseline clinical measures.

Variable	LUNAR NR (*n* = 9)	LUNAR CR (*n* = 5)	Abatacept NR (*n* = 19)	Abatacept CR (*n* = 15)	MUSC CR (*n* = 6)	Cohort Comparisons		All NR (*n* = 28)	All CR (*n* = 26)	All NR vs. CR *p*-Value
						NR *p*-Value	CR *p*-Value			
Age, years, mean (SD)	31.1 (13.0)	24.4 (5.9)	31.2 (5.8)	35.4 (7.9)	32.0 (8.5)	0.978	0.011	31.2 (8.54)	32.5 (8.57)	0.573
Race, *n* (%)						<0.001	<0.001			0.031
Black	1	2		1	6			1 (3.57)	9 (34.6)	
Hispanic	5	3						5 (17.86)	3 (11.5)	
Other/Asian			12	7				12 (42.86)	7 (26.9)	
White	3		7	7				10 (35.71)	7 (26.9)	
LN Class, *n* (%)						1.000	1.000			0.032
I					1			0. (0.00)	1 (3.85)	
III, IV	6	5	13	13	2			19 (67.9)	20 (71.4)	
III + V, IV + V	3		6	2				9 (32.1)	2 (7.14)	
V					2			0 (0.00)	2 (7.14)	
no biopsy					1			0 (0.00)	1 (3.85)	
C3 Comp, mean (SD)	72.6 (29.3)	75.3 (18.4)	56.6 (21.4)	70.7 (22.2)	59.9 (22.2)	0.142	0.683	62.0 (25.0)	69.1 (29.1)	0.270
C4 Comp, mean (SD)	12.9 (8.2)	11.4 (4.7)	13.7 (5.6)	15.3 (6.7)	9.9 (2.6)	0.373	0.151	13.4 (6.43)	14.34 (6.43)	0.624
Anti-dsDNA, median (IQR)	66.3 (322.1)	66.4 (1009.5)	89.8 (256.7)	76.6 (189.0)	174.0 (167.0)	0.797	0.617	71.9 (249.1)	94.0 (168.2)	0.560
UPr:UCr, mean (SD)	3.0 (2.6)	2.3 (1.8)	3.4 (2.6)	1.4 (1.6)	1.5 (1.9)	0.606	0.097	3.28 (2.56)	1.59 (1.66)	0.006
eGFR, mean (SD)	67.5 (34.2)	125.4 (17.4)	88.6 (33.1)	102.3 (23.2)	122.5 (26.9)	0.116	0.059	81.8 (34.3)	111.4 (24.8)	<0.001
Serum Creatinine, mean (SD)	1.3 (0.7)	0.7 (0.2)	1.0 (0.5)	0.8 (0.3)	0.7 (0.2)	0.099	0.431	1.1 (0.57)	0.75 (0.24)	0.005

Urine samples obtained from LN patients in the placebo arm of two clinical trials, LUNAR and abatacept, and from the biorepository at the Medical University of South Carolina (MUSC). *p*-values for the comparisons of “All NR vs CR” across all cohorts and “Cohort Comparisons” by response are based on a two-sample t-test for variables reporting mean (SD), a Wilcoxon rank-sum test for variables reporting median (IQR), and Fisher’s exact test for all categorical variables. Comp, complement; UPr:UCr, urine protein to urine creatinine ratio; eGFR, estimated glomerular filtration rate; SD, standard deviation; IQR, interquartile range.

**Table 2:** Baseline HexCers and LacCers (pmol/mg UCr) univariate analysis.

Marker	Non-Responders (*n* = 28) Median (IQR; min, max)	Complete Responders (*n* = 26) Median (IQR; min, max)	*p*-Value
HexCer C16	1.52 (1.70; 0.04, 9.79)	0.29 (0.52; 0.01, 2.36)	<0.001
HexCerC18	0.13 (0.29; 0.00, 1.37)	0.03 (0.08; 0.00, 0.23)	<0.001
HexCer C20	0.39 (0.92; 0.05, 4.14)	0.08 (0.18; 0.00, 0.70)	<0.001
HexCer C22:1	0.09 (0.14; 0.02, 1.68)	0.04 (0.08; 0.00, 0.18)	0.003
HexCer C22	0.93 (1.97; 0.19, 8.80)	0.29 (0.57; 0.02, 2.11)	<0.001
HexCer C24:1	0.92 (2.02; 0.13, 11.60)	0.17 (0.39; 0.01, 2.26)	<0.001
HexCer C24	1.03 (1.74; 0.08, 11.40)	0.26 (0.42; 0.01, 1.97)	<0.001
HexCer Total	5.33 (8.67; 0.56, 44.80)	1.21 (1.69; 0.07, 9.23)	<0.001
LacCer C16	4.63 (6.12; 0.11, 19.00)	0.71 (1.49; 0.00, 5.44)	<0.001
LacCer C18	0.21 (0.36; 0.00, 1.01)	0.04 (0.09; 0.00, 0.28)	<0.001
LacCer C20	0.15 (0.32; 0.00, 0.88)	0.03 (0.08; 0.00, 0.20)	<0.001
LacCer C22:1	0.10 (0.18; 0.00, 0.36)	0.03 (0.06; 0.00, 0.20)	0.001
LacCer C22	0.88 (1.31; 0.01, 3.09)	0.18 (0.32; 0.00, 1.59)	<0.001
LacCer C24:1	4.52 (5.89; 0.06, 16.2)	0.55 (1.55; 0.02, 6.29)	<0.001
LacCer C24	0.82 (1.17; 0.06, 4.06)	0.24 (0.44; 0.01, 1.69)	<0.001
LacCer Total	12.90 (15.60; 0.26, 44.20)	1.85 (3.85; 0.04, 15.10)	<0.001

The major hexosylceramides (HexCer) and lactosylceramides (LacCer) individual chain lengths (C16-C24) and a total of all chain lengths (Total) were measured by SFC/MS/MS, normalized to urine creatinine (UCr), and analyzed by univariate analysis. Univariate associations between GSL levels and treatment response were evaluated by Wilcoxon rank-sum tests. *p*-values were Bonferroni adjusted for multiple comparisons.

**Table 3:** AUC for baseline HexCers and LacCers.

Marker	AUC	*p*-Value
HexCer C16	0.88	0.011 *
HexCer C18	0.86	0.009 *
HexCer C20	0.89	0.003 *
HexCer C22:1	0.85	0.069
HexCer C22	0.87	0.011 *
HexCer C24:1	0.90	0.001 *
HexCer C24	0.87	0.015 *
HexCer Total	0.89	0.003 *
LacCer C16	0.88	0.012 *
LacCer C18	0.87	0.023 *
LacCer C20	0.85	0.106
LacCer C22:1	0.84	0.178
LacCer C22	0.87	0.147
LacCer C24:1	0.87	0.037 *
LacCer C24	0.87	0.039 *
LacCer Total	0.88	0.022 *

The major hexosylceramides (HexCer) and lactosylceramides (LacCer) individual chain lengths (C16-C24) and a total of all chain lengths (Total) were measured in all samples (Table 1) by SFC/MS/MS and normalized to urine creatinine (UCr) in the same sample. The area under the receiver operating characteristic curves (AUC) were estimated from a multivariate logistic regression adjusted for baseline UPr:UCr and eGFR. The *p*-values are the Bonferroni adjusted *p*-values based on a likelihood ratio test (LRT) comparing models with only UPr:UCr and eGFR to models that also include specific GSLs. * significant increase in AUC.

**Table 4:** Baseline Protein Univariate Analyses.

Marker	Non-Responders Median (IQR; min, max)	Complete Responders Median (IQR; min, max)	*p*-Value
EV Gelsolin	461.2 (779.1; 0.00, 3564)	490.2 (697.7; 0.0, 1627)	1.000
Urine Gelsolin	440.0 (561.0; 0.00, 2018)	126.0 (212.6; 17.4, 729.4)	<0.001 *
Urine LGALS3BP	132.0 (222.6; 1.0, 1880)	27.8 (225.9; 0.0, 455.5)	0.237
Eotaxin2	9.1 (12.3; 1.6, 59.6)	7.2 (8.1; 0.6, 37.3)	1.000
MCP2	8.1 (26.8; 0.0, 71.8)	5.3 (5.2; 0, 18.6)	1.000
BCA1	1.0 (1.7; 0.2, 9.4)	0.4 (0.4; 0.2, 4.2)	1.000
IL16	17.5 (19.1; 0.2, 46.0)	3.6 (7.2; 0.0, 21.7)	0.237
6CKine	0.0 (19.9; 0.0, 255.3)	0.0 (7.6; 0, 18.3)	1.000
TPO	62.9 (91.2; 12.9, 901.6)	34.2 (26.1; 13.0, 218.3)	1.000
SCF	13.5 (14.4; 0.3, 50.9)	11.1 (16.9; 2.4, 49.7)	1.000
TSLP	1.2 (8.26; 0.1, 51.0)	0.7 (0.6; 0.0, 1.14)	0.189
IL33	4.94 (18.7; 0.3, 120.3)	3.1 (1.6; 0.7, 11.5)	0.823
IL20	80.1 (131.5; 0.0, 2496.2)	52.8 (55.1; 0, 133.4)	1.000
IL23	60.5 (98.8; 1.6, 1737.5)	21.4 (29.8; 0.0, 58.4)	0.537
CTACK	1.1 (1.2; 0.0, 21.0)	0.4 (0.2; 0.0, 0.9)	0.108
SDF1 a + b	70.0 (352.1; 0.0, 1522.1)	61.0 (65.7; 0.0, 184.9)	1.000
ENA78	9.0 (20.3; 0.0, 149.4)	6.5 (5.9; 0.0, 14.2)	1.000
MIP1d	48.9 (73.3; 0.0, 368.7)	21.3 (28.1; 0.0, 59.5)	0.946

Proteins were measured as described in the Section 4, normalized to urine creatinine (UCr) measured in the same sample, and analyzed by univariate analysis. Gelsolin was measured in both urine extracellular vesicles (EV) and whole urine (Urine). Levels of gelsolin and LGALS3BP were measured in all available patient samples, while urine chemokines were measured in 15 patients with class III or IV LN only (see Figure S1). Univariate associations between protein levels and treatment response were evaluated by Wilcoxon rank-sum test and *p*-values were adjusted for multiple comparisons by Bonferroni. LGALS3BP, galectin-3 binding protein; MCP2, monocyte chemotactic protein 2; BCA1, B cell-attracting chemokine 1; IL16, lymphocyte chemoattractant factor (LCF); 6CKine, C-C Motif Chemokine Ligand 21 (CCL21); TPO, thyroid peroxidase; SCF, stem cell factor; TSLP, thymic stromal lymphopoietin; CTACK, cutaneous T cell-attracting chemokine; SDF1 α + β, stromal cell-derived factor 1 alpha and beta; ENA78, epithelial-derived neutrophil-activating protein 78; MIP1δ, macrophage inflammatory protein 1 delta. * significant difference between NR and CR.

## Data Availability

Data is contained within the article or as Supplementary Materials.

## Supplementary Materials

The following are available online at https://www.mdpi.com/article/10.3390/metabo12020134/s1, Figure S1: Workflow describing sample origin and numbers, urine EV isolation, and analyses performed, Figure S2: Total HexCers and LacCers levels are significantly higher in baseline urine EVs of patients that did not respond to therapy, Figure S3: All chain lengths of HexCers and LacCers are significantly higher in baseline urine EVs of patients that did not respond to therapy, Figure S4: Volcano plot of proteins identified in proteomics screen of baseline urine EVs, Figure S5: Urine chemokines at baseline and 3 months post-treatment in patients with class III and IV LN only, Table S1: Baseline univariate analyses excluding class I, V, and no biopsy patients, Table S2: Baseline univariate analyses of pure class III and IV patients only.
