# Supplementary material for: Glycosphingolipid Levels in Urine Extracellular Vesicles Enhance Prediction of Therapeutic Response in Lupus Nephritis
Source: Metabolites. 2022 Feb 1;12(2):134. doi: 10.3390/metabo12020134 (PMC8876142; doi:10.3390/metabo12020134)
Supplement: Supplementary file 1 [file metabolites-12-00134-s001.zip › metabolites-1488480-supplementary.pdf]

Supplementary Materials

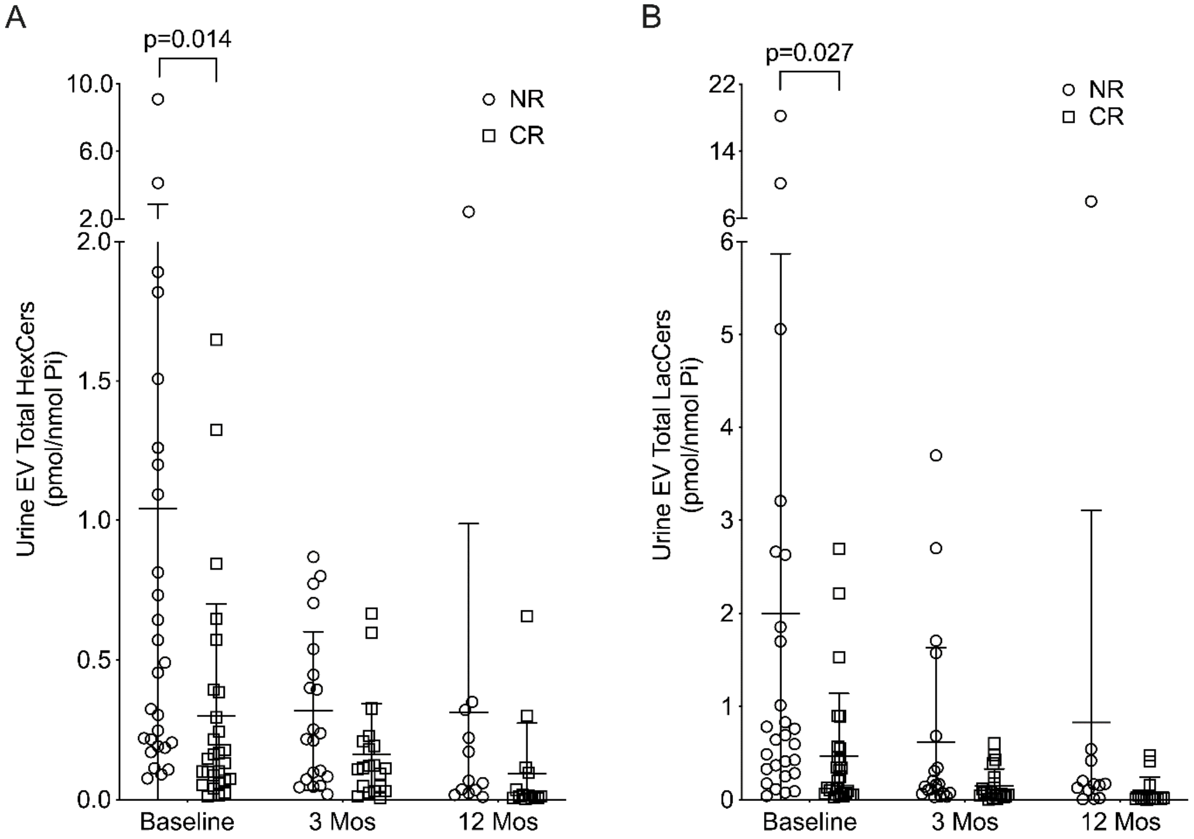

Figure S1. Total HexCers and LacCers are significantly higher in baseline urine EVs of patients that did not respond to therapy. EVs were isolated from urine samples of LN patients that me the clinical criteria of a non-responder (NR) or a complete responder (CR) after 12 months of treatment with MMF. Total of all chain lengths of HexCers (A) and LacCers (B) were quantified in EVs from urine collected prior to treatment (baseline) and at 3 months (3 Mos) and 12 Months (12 Mos) posttreatment. HexCers and LacCers were normalized to inorganic phosphate (Pi) levels in the EVs. Error bars represent standard deviation. P-values were calculated as described in Materials and Methods and in the Results.

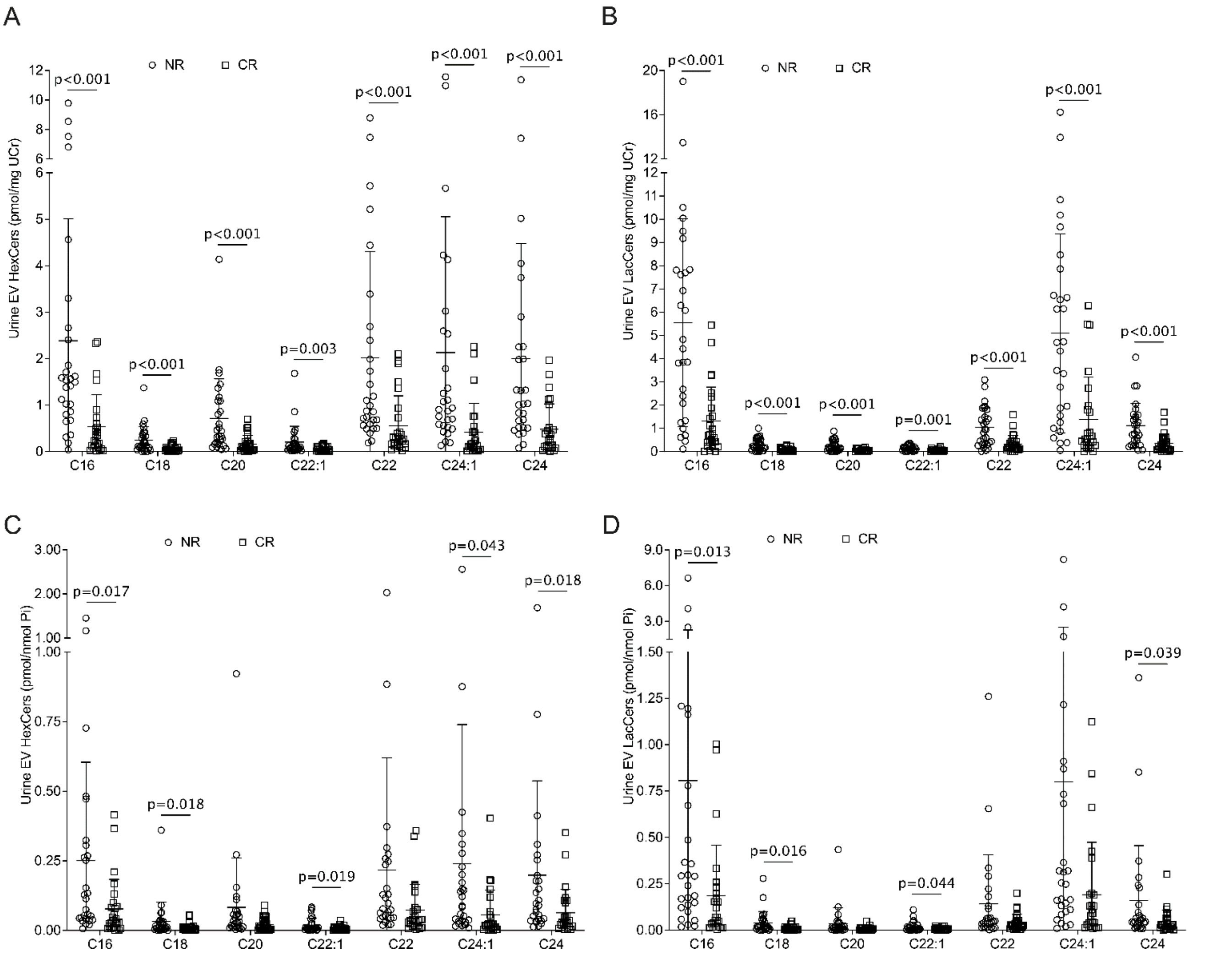

Figure S2. All chain lengths of HexCers and LacCers are significantly higher in baseline urine EVs of patients that did not respond to therapy. EVs were isolated from urine samples of LN patients that met the clinical criteria of a non-responder (NR) or a complete responder (CR) after 12 months of treatment with MMF. Total of all chain lengths of HexCers (A and C) and LacCers (B and D) were quantified in EVs from urine collected prior to treatment (baseline) and at 3 months (3 Mos) and 12 months (12 Mos) post-treatment. HexCers and LacCers were normalized to UCr (A and B) or inorganic phosphate (Pi) levels in the EVs (C and D). Error bars represent standard deviation. P-values were calculated as described in Materials and Methods and in the Results.

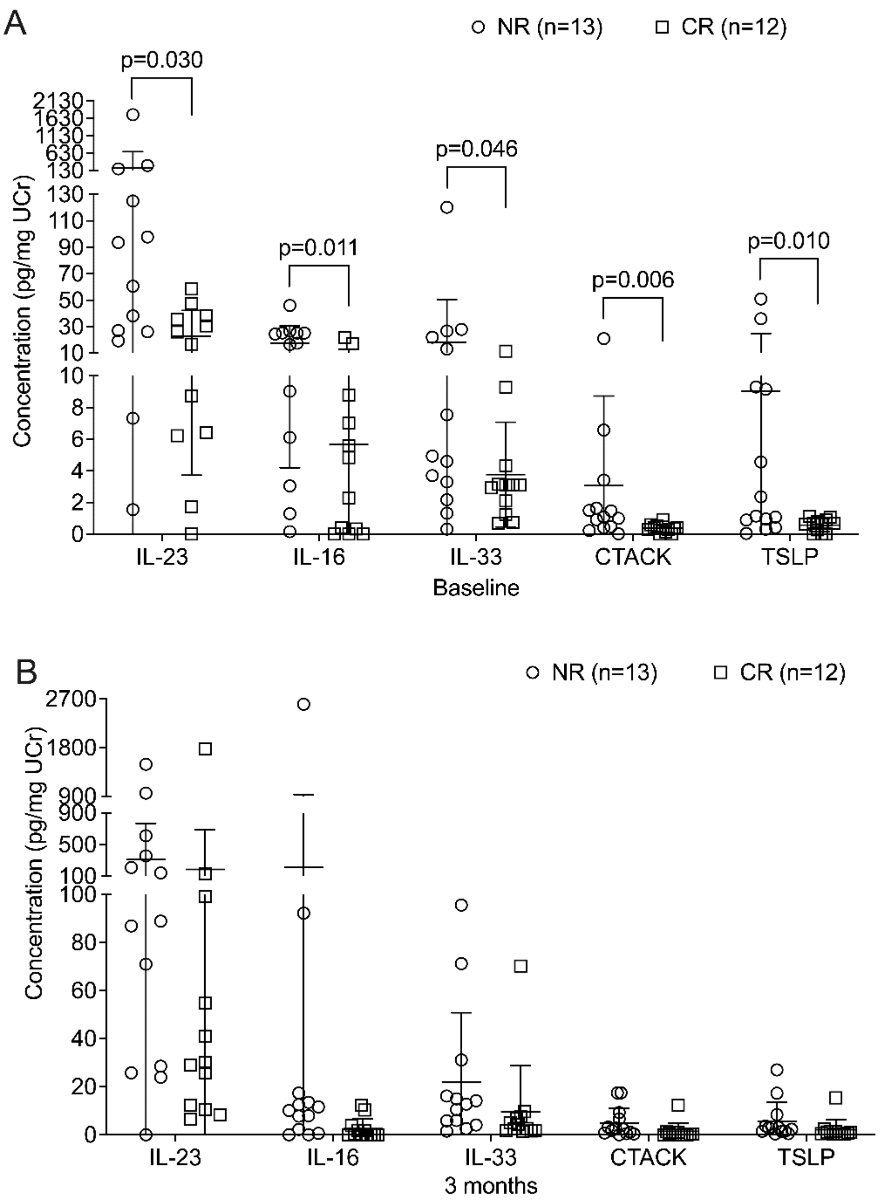

Figure S3. Several Chemokines are significantly higher in baseline urine of patients that did not respond to therapy. Levels of 23 chemokines were quantified in baseline (A) and 3 months (B) whole urine samples from a subset of the LN patients in Figure 1. All measures were normalized to UCr. Error bars represent standard deviation. P-values were calculated as described in Materials and Methods and in the Results.
